# Supplementary material for: Healthcare worker perspectives on adaptations to differentiated anti-retroviral therapy delivery during COVID-19 in South Africa: A qualitative inquiry
Source: PLOS Glob Public Health. 2024 Aug 9;4(8):e0003517. doi: 10.1371/journal.pgph.0003517 (PMC11315304; doi:10.1371/journal.pgph.0003517)
Supplement: S1 File — (PDF) [file pgph.0003517.s003.pdf]

**ASSESSING IMPLEMENTATION OF DIFFERENTIATED CARE ADAPTATIONS TO HIV SERVICES DURING COVID-19 (AIDAH)**

**Participants Semi-Structure In-Depth Interview Flexible Topic Guide**

Thank you for taking the time to talk with me today. I am looking forward to hearing your thoughts and opinions about how the CCMDD programme has worked during COVID-19.

As a reminder, you are not required to answer all my questions if you feel uncomfortable answering them.

The discussion should last between half an hour to an hour. You are the expert, and so I am eager to hear what you have to say. There are no "right" answers – just your own experiences and thoughts.

Do you have any questions before we begin the discussion?

**Note to the interviewer: Turn on a digital recorder if consent has been provided.**

I am [INTERVIEWERS NAME], interviewing PID-[IDI#] on [DATE] [START TIME]

| MAIN QUESTION | PROBES |
|---|---|
| **INTRODUCTION** | |
| Can you tell me a bit about yourself and your primary role at work? | And your involvement with CCMDD? |
| **COVID-19 ADAPTATIONS TO CCMDD** | |
| Can you tell me about your work with the CCMDD programme since the beginning of the COVID-19 pandemic? | How has your work changed?<br><br>The Department of Health introduced some changes to CCMDD during COVID-19, can you tell me about these?<br><br>Which changes worked well?<br><br>Which changes did not work well? |
| | |
| The CCMDD programme relies on a lot of different systems and processes to make sure patients can get their treatment.<br><br>How were these systems adapted to allow the COVID-19 changes to be made? | For example, processes at the clinics and the SYNCH system?<br><br>At the pick-up points?<br><br>From the service provider who delivers the treatment.<br><br>At the telephone helpdesk for patients?<br><br>How do these different processes fit together?<br><br>How did these changes affect the reliability of the CCMDD programme? |

| | |
|---|---|
| | What are your thoughts around the usability of the CCMDD system after these adaptations? |
| | |
| How have these adaptations changed how you provide healthcare in your facility? | Has this affected your work positively or negatively? And why?<br><br>How about other people working in CCMDD (Doctors, Nurses, Pharmacists)<br><br>What impact do you think these adaptations have on this facility?<br><br>How would you say these adaptations are impacting the healthcare system?<br><br>What do patients think about these changes? |
| | |
| How prepared was this facility when these adaptations were introduced? | Was training provided to staff when these adaptations were launched?<br><br>Were there key people (such as senior management) who opposed these adaptations?<br><br>What did they say? |
| | |
| What challenges do you think will make this adaptation not sustainable in the long run? | |
| | |
| How cost-effective do you think these adaptations are? | To the facility<br>The Health Department |
| | |
| There are many different organisations involved in CCMDD. What do you think these other structures such as pick-up-points and pharmaceutical companies support or oppose these adaptations? | Did they have any concerns about them? If so, what are they?<br><br>-Quality/safety care?<br>-Workload?<br>-Other pressing priorities? |
| | |
| Were patients with other health conditions taken into considering when these adaptions were launched? | Did they have to use the healthcare facility to access other healthcare services apart from HIV care? |
| **Wrapping up** | |
| Were there any other changes regarding CCMDD that were implemented because of the COVID pandemic? | |
| In your opinion, would you suggest these changes remain in the future? Why? | |
| Is there anything else you would like to add regarding CCMDD, HIV care or COVID-19? | |
