# Supplementary material for: Healthcare worker perspectives on adaptations to differentiated anti-retroviral therapy delivery during COVID-19 in South Africa: A qualitative inquiry
Source: PLOS Glob Public Health. 2024 Aug 9;4(8):e0003517. doi: 10.1371/journal.pgph.0003517 (PMC11315304; doi:10.1371/journal.pgph.0003517)
Supplement: Attachment — Submitted filename: Response to Reviewers.docx [file pgph.0003517.s005.docx]

Henry Zakumumpa

Academic Editor

PLOS Global Public Health

1265 Battery Street Suite 200

San Francisco, CA 94111

United States of America

08 July 2024

Dear Dr. Zakumumpa and Colleagues,

Thank you for presenting us with detailed reviews of our manuscript, “Healthcare worker perspectives on adaptations to differentiated antiretroviral therapy delivery for people living with HIV during COVID-19 in South Africa” and for inviting the revision and resubmission of our work. Taking the reviewers’ and editorial comments into account, we have carefully revised the manuscript. Please find below a point-by-point response to the comments. Additions or changes made in the manuscript in response to the reviewers’ comments have been bolded. Line numbers are for the clean copy of the revised manuscript.

**JOURNAL REQUIREMENTS**

Requirement 1: Please include a complete copy of PLOS’ questionnaire on inclusivity in global research in your revised manuscript.

Response**:** A copy of the PLOS’ questionnaire on inclusivity in global research has been uploaded under Supporting information and an indication of completion of the questionnaire has been included in the revised manuscript under Supporting Information as ***‘S3 File: PLOS’ questionnaire on inclusivity in global research’*** *(line 652, page 26).*

Requirement 2: Please review your reference list to ensure that it is complete and correct.

Response: We thank the editor for this request, we have reviewed our reference list and have ensured that it is complete and correct. The date cited has been added to the web page citation used in the manuscript:

South African Government. Official Guide to South Africa 2021/22: Government Communication and Information System 2022 **[cited 2024 23 February]**. 1-6]. Available from: <https://www.gov.za/about-sa/people-south-africa-0> *(References, line 567-569, page 23).*

Joint United Nations Programme on HIV/AIDS (UNAIDS). UNAIDS DATA 2022. Joint United Nations Programme on HIV/AIDS Geneva2022 **[cited 2023 07 November]**. Available from: <https://www.unaids.org/sites/default/files/media_asset/data-book-2022_en.pdf> *(References, line 570-572, page 23).*

Ho U. COVID-19: Some chronic patients to be given enough pills for 3 or 4 months2020 **[cited 2023 30 August]**. Available from: <https://www.spotlightnsp.co.za/2020/03/27/covid-19-some-chronic-patients-to-be-given-enough-pills-for-3-or-4-months/> *(References, line 594-596, page 24).*

South African National Department of Health. Response to reduce risk among HIV and TB patients within the context of the COVID‐19 pandemic: The South African response to COVID‐19. 2020, 17 April **[cited 2023 22 November]**. 1–13]. Available from: <https://www.covidlawlab.org/wp-content/uploads/2020/06/South-Africa_COVID-DSD_2020_Reduce-risk-among-HIV-and-TB-patients.pdf> *(References, line 625-629, page 25).*

South African National AIDS Council. The National Strategic Plan for HIV, TB, and STIs 2023-2028.2023, March. **[cited 2024 23 February]**. Available from: <https://knowledgehub.health.gov.za/system/files/elibdownloads/2023-04/NSP-HIV-TB-STIs-2023-2028-MARCH20_23-PRINT2.pdf> *(**References, line 636-646, page 25).*

**REQUIREMENT REGARDING DATA SHARING (SENT ON 06 JULY 2024)**

Comment: a) We note that you have indicated that data from this study are available upon request. PLOS only allows data to be available upon request if there are legal or ethical restrictions on sharing data publicly… In line with our goal of ensuring long-term data availability to all interested researchers, PLOS’ Data Policy also states that authors cannot be the sole named individuals responsible for ensuring data access.

i. If there are ethical or legal restrictions on sharing a de-identified data set, please explain the relevant restrictions in detail (e.g., data contain potentially identifying or sensitive patient information) and who has imposed them (e.g., a Research Ethics Committee or Institutional Review Board, etc.).
ii. Please provide non-author contact information (phone/email/hyperlink) for a data access committee, ethics committee, or other institutional body to which data requests may be sent.
iii. If applicable, please also provide any necessary information which interested researchers would need when requesting access to data in order to obtain the minimal data set for your study.

Response: We cannot publicly share the interview transcripts which this study was based on because interview transcripts may contain information that allows participants to be identified. In particular, healthcare workers with specific roles at relatively few clinics were interviewed in this study, and so other staff members may be able to identify participants based on the contents of transcripts, even if these do not contain directly identifiable information. Therefore, the consent form and study protocol, which have been approved by the Biomedical Research Ethics Committee of the University of KwaZulu-Natal, do not allow for public sharing of transcripts. Instead, in the interest of transparency, the protocol and consent form state that data can be shared with other researchers. We now provide amended details in the data access statement of how interested researchers can request access to the data:

***Researchers wanting to access data from this study are requested to complete a CAPRISA data request form. The form can be accessed from the link below. The written request will be assessed by the CAPRISA Scientific Review Committee within 30 business days, and once approved, the dataset will be made available. In line with standard data access principles, CAPRISA will ensure that metadata on the datasets will be made available and anonymization and other measures will be taken to protect individual and personally identifiable information in the datasets.***

[***https://www.caprisa.org/DBFile/Files/391bc205-fb20-4c3a-a4c5-57010e433697/REQUEST%20TO%20ACCESS%20DATA%20FROM%20A%20CAPRISA%20TRIAL.docx***](https://www.caprisa.org/DBFile/Files/391bc205-fb20-4c3a-a4c5-57010e433697/REQUEST%20TO%20ACCESS%20DATA%20FROM%20A%20CAPRISA%20TRIAL.docx) *(Data Availability statement, line 556-565, page 22).*

**RESPONSE TO REVIEWER 1**

Comment 1: Title: I advise that you re-word your title as "Healthcare worker perspectives on adaptations to differentiated anti-retroviral therapy delivery Model in South Africa: A Qualitative Inquiry" The study group, such as people living with HIV during COVID-19, can be described in the methodology.

Response**:** We appreciate the reviewer’s advice on the title. We have re-worded our title to ***“Healthcare worker perspectives on adaptations to differentiated anti-retroviral therapy delivery during COVID-19 in South Africa: A Qualitative Inquiry”*.** We believe it is crucial to specify in the title that the adaptations occurred during COVID-19 because some of these adaptations were COVID-19 specific and have been rescinded after the pandemic. Thus, we have retained this *(Title, page 1).*

Comment 2: Study Design: Read more about qualitative studies designs and revise your design with a more appropriate qualitative design.

Response**:** We thank the reviewer for this comment, we have revised the study methods section, including the design, setting and participants:

***Study Design***

***We used qualitative methods and employed an exploratory research design, informed by the interpretivist paradigm. This approach was chosen to understand the experiences and perspectives of HCWs in implementing adaptations to the CCMDD programme and the overall impact of COVID-19 on the programme.*** ***The reporting of the research processes in this study was guided by the Consolidated Criteria for Reporting Qualitative Research (COREQ) checklist*** *(Methods, line74-79, page 5).*

***Study setting and participants***

***The study was conducted in eThekwini District, KwaZulu-Natal, South Africa. We recruited healthcare workers, clinic operational managers and members of key stakeholder organisations purposively, based on their involvement in CCMDD, from nine public health clinics (eThekwini municipal clinics) and two key stakeholder organisations, such as HIV treatment non-governmental organisations (NGO) or community organisations, in the same district. The clinics were purposively selected based on their involvement in CCMDD, to ensure that interviewed participants had relevant experience and insights regarding the implementation of CCMDD adaptations and the impact of COVID-19 on the programme. Additionally, some clinics were included because they were the workplaces of potential participants referred by study participants using the snowball sampling technique(16). Participants were ≥18 years, spoke English or IsiZulu, and were willing to provide consent*** *(Methods, line 81-94, page 6).*

Comment 3: Results: Kindly summarize your themes and sub-themes in the background or introduction before using them in the body of your results.

Response**:** Thank you for this comment. However, we cannot summarize the themes and subthemes in the introduction in this study because they were inductively derived from the results, we therefore provided a summary at the beginning of the results and then discussed them in the discussion.

Comment 4: Data sharing: Write a statement on data sharing and show the link where your data may be found. You can deposit your dataset in a public repository such as Mendeleys, Harvard Dataverse and share the link with the readers. Transparency in research is very important and must be upheld.

Response: We thank the reviewer for this comment. However, due to the qualitative nature of our study, we cannot store the data in any public database as it can potentially have information in that could compromise participant confidentiality. Furthermore, participants did not consent to the transcripts being made publicly available. Instead, in the interest of transparency, researchers can request to access the data and we will ensure that data shared is responsive to a particular data query, and that all information is adequately de-identified in response to the query before being shared. We have included the following statement in the manuscript:

***Data Availability Statement***

***Researchers wanting to access data from this study are requested to complete a CAPRISA data request form. The form can be accessed from the link below. The written request will be assessed by the CAPRISA Scientific Review Committee within 30 business days, and once approved, the dataset will be made available. In line with standard data access principles, CAPRISA will ensure that metadata on the datasets will be made available and anonymization and other measures will be taken to protect individual and personally identifiable information in the datasets***

[***https://www.caprisa.org/DBFile/Files/391bc205-fb20-4c3a-a4c5-57010e433697/REQUEST%20TO%20ACCESS%20DATA%20FROM%20A%20CAPRISA%20TRIAL.docx***](https://www.caprisa.org/DBFile/Files/391bc205-fb20-4c3a-a4c5-57010e433697/REQUEST%20TO%20ACCESS%20DATA%20FROM%20A%20CAPRISA%20TRIAL.docx) *(Data Availability statement, line 556-565, page 22).*

**COMMENTS WITHIN THE MANUSCRIPT**

Comment 5: How were the HCWs chosen?

Response: We appreciate the query from the reviewer, we have provided a description under ‘Study setting and participants’ in the methods section:

*The study was conducted in eThekwini District, KwaZulu-Natal, South Africa****. We recruited healthcare workers, clinic operational managers and members of key stakeholder organisations purposively, based on their involvement in CCMDD****, from nine public health clinics (eThekwini municipal clinics) and two key stakeholder organisations, such as HIV treatment non-governmental organisations (NGO) or community organisations, in the same district (Methods, line 82-86, page 6).*

Comment 6: How were the clinics chosen?

Response: We appreciate the query from the reviewer, we have provided a description under ‘Study setting and participants’ in the methods section:

***The clinics were purposively selected based on their involvement in CCMDD, to ensure that interviewed participants had relevant experience and insights regarding the implementation of CCMDD adaptations and the impact of COVID-19 on the programme. Additionally, some clinics were included because they were the workplaces of potential participants referred by study participants using the snowball sampling technique*** *(Methods, line 88-94, page 6).*

Comment 7: Define theoretical saturation, better still use citation.

Response: We thank the reviewer for this comment, we have defined theoretical saturation with the use of a citation under ‘Data collection’ in the methods section:

*We aimed for 20-40 interviews but stopped at 21 when theoretical saturation was reached****, meaning no new themes or insights emerged (17)*** *(line 104-105, page 6).*

**RESPONSE TO REVIEWER 2**

Comment: I believe this is an original research. The motive is important and timely. The researchers identified key themes that related to their original quest. The analysis was appropriately performed and findings presented in a logical and understandable manner. The arguments are logical. Overall, the paper is chronological, the English grammar of the paper is fair and ethics aspects was well considered.

Response: We thank the reviewer for the commendation.

Kindest regards,

Kwena Tlhaku (She/Her)

**PhD Research Fellow**

**Centre for the AIDS Programme of Research in South Africa (CAPRISA)**

**University of KwaZulu-Natal**
